# An inverse association of weight and the occurrence of asymptomatic gallbladder stone disease in hypercholesterolemia patients: a case-control study

**DOI:** 10.1186/s12944-020-01402-8

**Published:** 2020-10-23

**Authors:** Binwu Sheng, Qingbin Zhao, Mao Ma, Jianqin Zhang

**Affiliations:** 1 Department of Geriatric Surgery, the First Affiliated Hospital of Xi’an Jiaotong University, 277 West Yanta Road, Xi’an, 710061 China; 2 Department of Geriatric, the First Affiliated Hospital of Xi’an Jiaotong University, 277 West Yanta Road, Xi’an, 710061 China; 3 Department of Nutrition, The Fourth People’s Hospital of Shaanxi, No. 512, Xianning East Road, Xi’an, 710043 China

**Keywords:** Gallbladder stone disease, Hypercholesterolemia, Overweight, Obesity, Nonalcoholic fatty liver disease, Gallbladder cancer, Epidemiology

## Abstract

**Background:**

Despite the fact that the majority of gallstones formed in the gallbladder are mainly composed of cholesterol, as they are formed from cholesterol-supersaturated bile, and hypercholesterolemia is a common metabolic disorder, which is closely related to cardiac, hepatic, renal and other oxidative damage inflammation and necrosis, there is still no consensus regarding the contribution of blood serum lipids in the pathogenesis of gallbladder stone disease (GSD). This study aimed to investigate the relationship between hypercholesterolemia and the risk of new-onset asymptomatic GSD, and to determine the prevalence of factors associated with new-onset asymptomatic GSD in patients with hypercholesterolemia.

**Methods:**

In this study, 927 Chinese patients with new-onset asymptomatic gallstone disease and 845 healthy controls were enrolled starting from August 2012. Patients were matched for age, gender, race, occupation, systolic blood pressure, diastolic blood pressure, and fasting blood glucose levels (FBG). Body mass index (BMI), nonalcoholic fatty liver disease (NAFLD) and serum lipids indexes were compared and the relationships between BMI, blood lipid and gallbladder stone hazards were examined by logistic multivariate regression models.

**Results:**

The result showed a significantly higher morbidity with GSD in hypercholesterolemia than non-hypercholesterolemia patients (Χ^2^ = 17.211, *P* < 0.001). Of hypercholesterolemia patients, low density lipoprotein (OR = 1.493, *P* = 0.029) and NAFLD (OR = 2.723, *P* = 0.022) were significant risk factors for GSD, while being male (OR = 0.244, *P* = 0.033), weight (OR = 0.961, *P* = 0.022), high density lipoprotein (OR = 0.305, *P* < 0.001), and FBG (OR = 0.687, *P* = 0.034) were significantly negatively correlated with GSD in univariate analysis. Multivariate logistic regression indicated weakly positive correlations with NAFLD (OR = 3.284, *P* = 0.054), and significant negative correlations with weight (OR = 0.930, *P* = 0.018), HDL-c (OR = 0.144, *P* < 0.001), and GSD.

**Conclusion:**

Hypercholesterolemia acts as an independent risk factor for new-onset asymptomatic GSD, while obesity and NAFLD are synergistic factors. Interestingly, it is first reported that elevated weight was inversely associated with GSD in patients with hypercholesterolemia. The results of this study suggest that effective control of hyperlipidemia is of greater significance than weight loss, which might make the situation worse, in the prevention of GSD in obese patients with hyperlipidemia.

## Background

Gallbladder stone disease (GSD) is a frequent problem in developed countries, representing a major health burden [1]. In the United States, approximately 10–20% of the national adult population currently carries gallstones, and the prevalence is rising. Nearly 750,000 cholecystectomies are performed annually. Cholelithiasis is strongly associated with gallbladder, pancreatic and colorectal cancer. The National Institutes of Health estimates that almost 3000 deaths (0.12% of all deaths) per year are attributed to complications of cholelithiasis and gallbladder disease. The direct and indirect costs of gallbladder surgery are estimated to be $6.5 billion [2]. With the westernization of the Chinese diet and environment, GSD is no longer rare among the Chinese population and has become a major health problem coincidentally [3]. Asymptomatic gallstones have become prevalent in the general population, imposing heavy economic burdens because of diagnosis, treatment, and indirect health care costs [4]. The prevalence of asymptomatic gallstones is 12.1% in China, with an increasing trend [5].

Although extensive research has tried to identify risk factors for GSD, several studies indicated that definitive findings remain inconclusive [6]. Traditionally, studies have suggested that cholesterol gallstone disease depends on a complex interplay between genetic factors, lifestyle and diet, acting on specific pathogenic mechanisms [7]. Until recently, it has been understood that cholesterol gallstone formation is mediated by genetic and environmental factors [8] and inflammation-mediated pathogenesis of cholesterol gallstones has not yet been considered seriously [9]. Overweight, obesity, dyslipidemia, insulin resistance and altered cholesterol homeostasis have been linked to increased gallstone occurrence, and are therefore modifiable by primary prevention measures related to diet, lifestyle, and environmental factors (including rapid weight loss, bariatric surgery, somatostatin or analogue therapy, transient gallbladder stasis and hormone therapy) [7, 10].

These studies have also shown that hyperlipidemia is associated with gallstones; nevertheless, the relationship between levels of serum lipids (blood cholesterol, triglyceride, low density lipoprotein, high density lipoprotein) and gallstones remains unclear. However, it is well known that cholesterol gallstones account for 80–95% of the gallstones found during cholecystectomy [11, 12], as they are formed from cholesterol-supersaturated bile [13]. Hypercholesterolemia is considered among the major risk factors for the progress of atherosclerotic vascular disease(i.e. ischemic heart disease), fatty liver, and kidney disease since it might induce lipid peroxidation, low GSH levels, and diminished even after activities of antioxidant enzymes, and eventually cause functional damage to the endothelium, consequently morphological lesions develop. Hypercholesterolemia, closely related to diabetes and obesity, may deposit fats and triglycerides into the liver, which usually leads to cirrhosis of the liver or even cellular liver cancer [14, 15]. In accordance with this viewpoint, another recent study showed that high levels of cholesterol were associated with gallbladder disorders such as cholesterolosis and gallstone disease, suggesting a positive association between hyperlipidemia and gallstones in humans [16].

In northwest China, many types of local foods are associated with specific ethnic groups. Recently, hyperlipidemia, obesity and GSD patients have been increasing yearly, coinciding with the westernization of the Chinese diet and increased population mobility. Studies showed that a high cholesterol diet might trigger hypercholesterolemia even after short term exposure. In contrast, dietary factors that may prevent the development of gallstones include polyunsaturated fat, monounsaturated fat, fiber, and caffeine [14]. This case-control study aimed to investigate the relationship between risk factors such as obesity and hypercholesterolemia with new-onset asymptomatic gallstone disease. The study reviewed the clinical records of patients with gallbladder stone during a 6-year period so as to determine whether hyperlipidemia was related to new-onset asymptomatic gallstone disease in a northwestern population in China. The findings in this study would hopefully shed new light on the etiology of GSD in the context of the current lifestyle.

## Methods

### Data source and study participants

927 GSD patients, aged 30 to 82 years, had undergone routine health check-ups annually in the healthcare center of the First Affiliated Hospital of Medical School of Xi’an Jiaotong University, from August 2012 to July 2018. They were diagnosed with new-onset gallbladder stone by abdominal ultrasonography examination in 2018. Ninety-two patients were excluded from the analysis for the following reasons: 1) Body mass index (BMI) < 18.5 Kg/m^2^; 2) history of gastrointestinal surgery, hepatobiliary disease, weight loss, or any cancer; 3) new diagnosis of nonalcoholic fatty liver disease (NAFLD) within 2 years; 4) BMI had not been maintained within the corresponding BMI category since 2012; i.e., BMI was elevated beyond the range of 24–27.99 Kg/m^2^, defined as overweight; 5) the data were missing in any of the following items: age, height, weight, systolic pressure (SBP), diastolic pressure (DBP), serum total cholesterol (TC), triglyceride (TG), low density lipoprotein cholesterol (LDL-c), high density lipoprotein cholesterol (HDL-c), fasting blood glucose (FBG), or ultrasonographic examination for gallbladder; and 6) future of matching failure in any following item: age, gender, race, occupation, diet including drinking, SBP, DBP and fasting blood glucose. A total of 825 healthy controls, who had undergone health check-ups annually in the same healthcare center since August 2012, were matched with subjects. This study was approved by the institutional review board and the committee of the First Affiliated Hospital of Medical School of Xi’an Jiaotong University and was conducted in accordance with the institutional ethics committee requirements. The need to obtain consent from the subjects was waived by the institutional review board (No.XJTU1AF2019LSK-017). The corresponding author and all co-authors had access to all data from this study and reviewed and approved of the manuscript.

Each subject was asked to complete a questionnaire and their demographic data and clinical indicators were collected. The questionnaire was designed by the authors and included the following items: telephone numbers, address, marital status, gravidity, oral contraceptive use, hypertension, history of diabetes mellitus, hyperlipidemia, gastrointestinal surgery, chronic liver disease, systemic diseases, family medical conditions, and other medications. Age was validated by their ID card.

In addition to completing the questionnaire, participants were asked to complete a screening panel including common cancer biomarkers, hypertension and transabdominal ultrasonography performed at the site. Blood samples were collected after a 10-h fast from an antecubital vein. These samples were used to assay serum TG, LDL-c, HDL-c, TC, FBG analyses were done using the glucose oxidase method from DiaSys Diagnostic Systems GmbH (Germany). All measurements were done in a central laboratory in a blinded fashion, and according to the manufacturer’s instructions. Blood pressure was measured three times by RBP 900 automatic blood pressure measuring instrument (Shenzhen Reycome Science and Technology Ltd. China), and the lowest figure would be available. Participants sat relaxed or had a rest for 30 min.

### Assessment and definitions

A trained clinician measured the height and weight of all participants using an HW-900Y ultrasonic wave height and weight scale (Jiangsu Hengfeng weighting, China) during the medical examination. Weight was measured without shoes and in light clothing to the nearest 0.1 kg. Height was determined to the nearest 0.1 cm without shoes. Waist circumference (WC) was measured using a flexible, tension-sensitive, non-stretching tape measure placed directly on the skin. Participants stood relaxed, with arms folded comfortably across the chest so multiple WC measurements could be more easily made. Measurements were made at the end of normal expiration with special attention paid to ensure the tape be positioned perpendicular to the long axis of the body and parallel to the floor. The same trained researcher took a series of four measurements from the right side of each subject by the previously published method [17]. Waist-to-hip ratio (WHtR) was calculated as WC in centimeters divided by height in centimeters and multiplied by a hundred (percentage, %). Body mass index (BMI) was calculated as weight in kilograms divided by squared height in meters (kg/m^2^). Patients were grouped by BMI according to expert consensus for medical nutrition therapy of overweight/obesity in China [18], where patients with BMI < 23.9 kg/m^2^ were considered normal, 24–27.9 kg/m^2^ considered overweight, and > 28 kg/m^2^ obese. High total cholesterol (hypercholesterolemia) was defined as a serum total cholesterol level of ≥6.22 mmol/L (240 mg/dL) or LDL-C ≥ 4.14 mmol/L (160 mg/dL) were diagnosed with hypercholesterolemia, based on blood lipoprotein profiles by the joint committee for developing Chinese guidelines on prevention and treatment of dyslipidemia in adults and The Adult Treatment Panel III [19–21].

A color Doppler ultrasonic instrument (Toshiba, SSA-510A, Japan) was used to perform to find gallbladder and hepatic diseases. The subjects were fasting and in the supine position. The liver, gallbladder, pancreas and spleen were examined in turn. If a hyperechoic mass with a clear acoustic shadow was seen in the gallbladder cavity, and the location of the mass changed with gravity, a gallstone was diagnosed. No intrahepatic or extrahepatic bile duct stones were found using B-mode ultrasonography.

Fatty liver was diagnosed if patients with the following items A + B and any of items C–F [22]: A, No history of alcohol overconsumption (< 210 g per week in men and < 140 g per week in women during the past 12 months). No long-term history of taking steatogenic medications, i.e., amiodarone, methotrexate, tamoxifen or glucocorticoids. The diseases that can lead to fatty liver such as genotype 3 HCV infection, Wilson’s disease, autoimmune hepatitis, total parenteral nutrition, hypo-β-lipoproteinemia, congenital lipodystrophy, and celiac disease were excluded; B, Echoes of diffuse enhancement were exhibited in the near-field of the hepatic region, and the decayed echo in the far-field; C, There were unclear echoes of intrahepatic duct; D, The liver was enlarged mildly to moderately and its edge angle was blunt; E, Color Doppler images suggested that the strikes of intrahepatic blood vessels were normal while the intrahepatic color blood flow signal were reduced or difficult to visualize; and; F, Unclear or incomplete echoes of the hepatic right lobe membrane and diaphragm were found.

### Statistical analysis

Descriptive data of participant characteristics were expressed as means ± standard deviations (SD) for continuous variables, and percentage (%) for categorical variables. Student’s t-test and the Chi square test was applied to compare continuous variables and categorical variables, respectively. The relationships between BMI, WC, WHtR, NAFLD, blood lipid and gallbladder stone hazards were examined by using logistic univariate and multivariate regression models, and were stratified by hypercholesterolemia. *P-*values < 0.05 were considered statistically significant. All statistical analyses were performed using SPSS version 19.0 software (SPSS Inc., Chicago, IL, USA).

## Results

### Characteristics of study participants

The characteristics and serum indexes in the gallbladder stone and control groups are summarized in Table 1. There were 835 of 927 patients with GSD and 835 control subjects of 3471 healthy participants aged 30 to 82 years. Each group included 322 (38.6%) female participants. There was significantly higher weight, BMI, WC, WHtR, serum TC, TG, LDL-c levels and lower HDL-c levels in the GSD group than in the controls (*P* < 0.05, respectively) (Table 1). There were 452 (54.1%) normal BMI, 319 (38.2%) overweight and 64 (7.7%) obese participants in the controls; in the GSD group, 331 (39.6%) had normal BMI, 386 (46.2%) were overweight and 118 (14.1%) were obese. There were 40 (5.4%) and 81 (10%) participants with hypercholesterolemia in the GSD and the control group, respectively. Statistical analysis showed significantly higher hypercholesterolemia in the GSD versus the control group (*P* < 0.001). In additional, there were 232 (27.5%) and 178 (21.1%) participants with NAFLD in the GSD and control groups, respectively. Statistical analysis showed significantly more NAFLD in the GSD group than in the control group (*P* = 0.002).

**Table 1:** Demographic and clinical data of the patients before and after matching

	Before matching				After matching			
	Control Group (*N* = 3471)	GSD group (*N* = 927)	χ^2^/T value	*P* value	Control group (*N* = 835)	GSD group (N = 835)	χ^2^/T value	*P* value
Female (%)	1713 (49.4%)	331 (35.7%)	55.537	< 0.001	38.6%, 322	38.6%,322	0.000^a^	1.000
Age (Year)	59.32 ± 13.7	59.14 ± 12.3	−6.041^a^	< 0.001	58.68 ± 11.4	58.65 ± 11.4	−0.060	0.952
Han (%)	2982 (85.9)	790 (85.2)	0.284^a^	0.594	760 (91.0)	765 (91.6)	0.189^a^	0.664
Occupation (%)			62.257^a^	< 0.001			0.476^a^	0.976
Worker	218 (6.3)	62 (6.7)	0.202^a^	0.653	62 (7.5)	60 (7.2)	0.027^a^	0.869
Professional Technician	316 (9.1)	112 (12.1)	7.043^a^	0.008	112 (13.5)	109 (13.1)	0.051^a^	0.821
Manager	193 (5.6)	81 (8.7)	11.693^a^	0.001	81 (9.7)	79 (9.5)	0.028^a^	0.868
Retiree	2062 (59.4)	583 (62.9)	3.729^a^	0.053	515 (61.9)	518 (62.0)	0.023^a^	0.880
Others	682 (19.6)	89 (9.6)	57.144^a^	< 0.001	62 (7.5)	69 (8.3)	0.406^a^	0.524
SBP (mmHg)	120.99 ± 17.9	122.70 ± 17.5	−2.586	0.01	122.05 ± 17.2	122.22 ± 16.2	0.209	0.834
DBP (mmHg)	76.76 ± 10.6	77.49 ± 10.77	−1.839	0.066	77.33 ± 10.5	77.10 ± 9.7	−0.465	0.642
FGB (mmol/L)	5.27 ± 1.3	5.18 ± 1.4	−0.677	0.499	5.38 ± 1.5	5.34 ± 1.2	−0.758	0.449
Height (cm)	165.27 ± 8.6	166.95 ± 8.2	−5.339	< 0.001	166.38 ± 7.2	166.71 ± 8.1	0.870	0.384
Weight (Kg)	65.82 ± 11.4	68.38 ± 11.2	6.053	< 0.001	66.77 ± 10.7	68.44 ± 10.9	3.170	0.002
BMI (Kg/m^2^)	24.00 ± 3.1	24.44 ± 2.9	0.905	< 0.001	24.04 ± 2.9	24.54 ± 2.8	3.580	< 0.001
Normal BMI(%)	1726 (49.7%)	431 (46.6%)	2.774^a^	0.096	452 (54.1%)	331 (39.6%)	35.332^a^	< 0.001
Overweight(%)	1126 (32.4%)	392 (42.4%)	31.477^a^	< 0.001	319 (38.2%)	386 (46.2%)	11.033^a^	0.001
Obesity(%)	619 (17.8%)	101 (10.9%)	27.441^a^	< 0.001	64 (7.7%)	118 (14.1%)	18.226^a^	< 0.001
WC (cm)	82.41 ± 7.0	83.53 ± 7.8	4.182	< 0.001	82.83 ± 6.7	87.75 ± 7.9	13.759	< 0.001
WHtR(%)	49.97 ± 4.7	49.82 ± 4.0	−6.768	< 0.001	49.84 ± 4.1	52.73 ± 5.0	12.827	< 0.001
TC (mmol/L)	4.88 ± 0.9	4.90 ± 1.0	0.610	0.542	4.65 ± 0.8	4.87 ± 0.9	5.329	< 0.001
TG (mmol/L)	1.49 ± 0.8	1.83 ± 1.3	9.911	< 0.001	1.51 ± 0.8	1.83 ± 1.2	6.120	< 0.001
HDL-c (mmol/L)	2.53 ± 1.0	1.28 ± 0.3	−35.764	< 0.001	2.25 ± 0.9	1.28 ± 0.3	−28.046	< 0.001
LDL-c (mmol/L)	2.0 ± 1.3	3.0 ± 0.8	22.215	< 0.001	2.00 ± 1.0	2.99 ± 0.8	21.502	< 0.001
Drink (%)	506 (13.5)	124 (13.4)	0.009^a^	0.925	105 (12.6)	107 (12.8)	0.022^a^	0.883
Eating habits (%)			117.822^a^	< 0.001			0.148^a^	0.986
Vegan	127 (3.7)	78 (8.4)	32.396^a^	< 0.001	69 (8.3)	71 (8.5)	0.031^a^	0.860
vegetarian	508 (14.6)	115 (12.4)	3.074^a^	0.080	114 (13.7)	110 (13.2)	0.082^a^	0.774
Meat & vegetarians	2730 (78.7)	544 (58.7)	142.746^a^	< 0.001	470 (56.3)	468 (56.0)	0.010^a^	0.921
Carnivorous diet	376 (10.8)	190 (20.5)	55.295^a^	< 0.001	182 (21.8)	186 (22.3)	0.056^a^	0.813
NAFLD	744 (21.4)	227 (24.5)	4.083^a^	0.043	178 (21.1)	232 (27.5)	9.448^a^	0.002
HC	327 (9.4)	106 (11.5)	3.343^a^	0.068	40 (4.8)	84 (10.1)	17.211^a^	< 0.001

*GSD* gallbladder stone disease; *SBP* systolic blood pressure; *DBP* diastolic blood pressure; *FBG* fasting blood glucose; *BMI* body mass index; *WC* waist circumference; *WHtR* Waist height ratio; *TC* total cholesterol; *TG* triglyceride; *HDL-c* high density lipoprotein cholesterol; *LDL-c* low density lipoprotein cholesterol; NAFLD: nonalcoholic fatty liver disease; HC: hypercholesterolemia

^a^means χ^2^ values for making a difference with t values

### The differences of hazard rate of gallbladder stone between patients with hypercholesterolemia and controls stratified by overweight and obesity or NAFLD

The differences in hazard rate of gallbladder stone in hypercholesterolemia and controls by obesity and overweight are summarized in Table 2. Among those with overweight and obesity, there were 39 and 15 participants with hypercholesterolemia in the GSD group, respectively, and 18 and 6 in the control group, respectively. There were significant differences in gallbladder stone morbidity between the hypercholesterolemia and control group in terms of overweight and normal BMI (χ^2^ = 4.813 and 10.424; *P* = 0.028, and 0.001, respectively), while there was no significant difference in obesity (χ^2^ = 0.466, *P* = 0.495).

**Table 2:** The difference of GSD between Hypercholesterolemia and non-hypercholesterolemia adjusted by overweight, obesity and normal BMI

	Overweight		Obesity		Normal BMI	
	GSD	Control	GSD	Control	GSD	Control
Model 1	39*	18	15 ^&^	6	30 ^**§**^	16
Model 2	347**	301	103 ^& &^	58	301 ^**§ §**^	436

**Note**:*GSD* gallbladder stone disease; *Model 1* Hypercholesterolemia; *Model 2*:Non-hypercholesterolemia

*vs **: χ^2^ = 4.813, *P* = 0.028, OR = 1.879, 95%CI: 1.053 ~ 3.355

^&^ vs ^&&^: χ ^2^ = 0.466, *P* = 0.495, OR = 1.408, 95%CI: 0.518 ~ 3.826

^**§**^vs^**§§**^: χ ^2^ = 10.424, *P* = 0.001, OR = 2.716, 95%CI: 1.455 ~ 5.070

As shown in Table 3, regarding NAFLD and non-NAFLD, there were 32 and 52 participants with hypercholesterolemia in the GSD group, respectively, and 9 and 31 in the control group, respectively. There was a significant difference regarding gallbladder stone morbidity between hypercholesterolemia and control groups in terms of NAFLD and non-NAFLD (χ^2^ = 9.157 and 7.839; *P* = 0.002 and 0.005, respectively).

**Table 3:** The difference of GSD between hypercholesterolemia and non adjusted by NAFLD and non-NAFLD

	NAFLD		non-NAFLD	
	GSD	Control	GSD	Control
Model 1	32*	9	52 ^&^	31
Model 2	200**	169	551 ^& &^	626

**Note**:*GSD* gallbladder stone disease group; *Model 1* Hypercholesterolemia; *Model 2* Non-hypercholesterolemia

*vs **^:^ χ^2^ = 9.157, *P* = 0.002, OR = 3.004, 95%CI: 1.395 ~ 6.472

^&^ vs ^&&^: χ ^2^ = 7.839, *P* = 0.005, OR = 1.906, 95%CI: 1.204 ~ 3.017

### Relationships between the indexes with BMI, WHtR, blood lipid, NAFLD, hyperlipidemia and hazard of cholesterol gallstone

Table 4 shows the risk factors for developing GSD based on univariate and multivariate logistic regression. Univariate logistic regression showed significantly positive correlations for weight, BMI, WC, WHtR, NAFLD, hypercholesterolemia, serum TC, TG and LDL-c levels with hazard of GSD (*P* < 0.05, all), while there were significantly negative correlations between serum HDL-c levels and risk of GSD (*P* < 0.001). In addition, compared with patients with normal BMI, there was significantly more GSD in overweight and obese patients (*P* < 0.001, both). Multivariate logistic regression including WC showed that there were significantly positive correlations between WC, NAFLD, hypercholesterolemia and risk of GSD (*P* < 0.05, all), while there were significantly negative correlations between serum HDL-c, TG levels, BMI and risk of GSD (*P* < 0.05, all). Multivariate logistic regression including WHtR showed that there was significantly positive correlations between WHtR, NAFLD, hypercholesterolemia, and risk of GSD (*P* < 0.05, all), while there were significantly negative correlations between serum TG, HDL-c levels, BMI and risk of GSD (*P* < 0.05, all).

**Table 4:** Univariate and multivariate analysis of gallbladder stone and BMI, WHtR, blood lipid, NAFLD and HTC

Factors		Univariate				Multivariate1				Multivariate2		
	β	OR	95% CI	*P*	β	OR	95% CI	*P*	β	OR	95% CI	*P*
Weight	0.013	1.013	1.004–1.022	0.004	*	*	*	*	*	*	*	*
BMI	0.044	1.045	1.010 ~ 1.081	0.011	−1.416	0.243	0.202 ~ 0.291	< 0.001	−0.518	0.596	0.544 ~ 0.652	< 0.001
Normal		1										
Overweight	0.125	1.652	1.346 ~ 2.029	< 0.001		/	/	/		/	/	/
Obesity	0.177	2.518	1.800 ~ 3.522	< 0.001		/	/	/		/	/	/
WC	0.077	1.080	1.065 ~ 1.095	< 0.001	0.517	1.677	1.571 ~ 1.790	< 0.001	/	/	/	/
WHtR	0.11	1.116	1.092 ~ 1.141	< 0.001	/	/	/	/	0.292	1.340	1.273 ~ 1.409	< 0.001
TG	0.323	1.382	1.237 ~ 1.543	< 0.001	−0.203	0.817	0.713 ~ 0.935	0.003	−0.205	0.814	0.721 ~ 0.919	0.001
HDL-c	−2.122	0.120	0.095 ~ 0.151	< 0.001	−1.965	0.140	0.103 ~ 0.191	< 0.001	−2.573	0.076	0.056 ~ 0.104	< 0.001
LDL-c	0.965	2.625	2.330 ~ 2.957	< 0.001	*	*	*	*	*	*	*	*
TC	0.251	1.286	1.150 ~ 1.437	< 0.001	*	*	*	*	*	*	*	*
FBG	−0.026	0.974	0.906–1.047	0.481	–	*–*	*–*	*–*	*–*	*–*	*–*	–
NAFLD	0.389	1.475	1.180 ~ 1.845	0.001	0.557	1.746	1.202 ~ 2.537	0.003	0.371	1.449	1.042 ~ 2.018	0.028
HC	0.786	2.195	1.502 ~ 3.208	< 0.001	0.874	2.397	1.398 ~ 4.112	0.001	0.872	2.392	1.456 ~ 3.929	0.001
SBP	0.001	1.001	0.996 ~ 1.007	0.67	–	–	–	–	–	–	–	–
DBP	0.000	1.000	0.991 ~ 1.010	0.919	–	–	–	–	–	–	–	–
Drink	0.082	1.085	0.813 ~ 1.448	0.579	–	–	–	–	–	–	–	–

*BMI* body mass index; *WC* waist circumference; *WHtR* Waist height ratio; *TG*: triglyceride; *HDL-c* high density lipoprotein cholesterol; *LDL-c* low density lipoprotein cholesterol; *NAFLD* nonalcoholic fatty liver disease; *FBG* fasting blood glucose; *HC* hypercholesterolemia; *SBP* systolic blood pressure; *DBP* diastolic blood pressure;

*eliminated for the definition of BMI or hyperlipidemia in multivariate regression

- eliminated for no statistical significance in univariate regression

To investigate the possible reasons that caused this result, the data were further analyzed using univariate and multivariate logistic regression analysis (Table 5). The differences between GSD and control group were studied by stratifying with hypercholesterolemia. Univariate logistic regression showed significantly positive correlations between NAFLD, serum LDL-c levels, and risk of GSD (*P* < 0.05 for all), high-level TC was a weak risk factor for GSD (*P* = 0.082), while there were significantly negative correlations for male, weight, DBP, serum HDL-c, FBG levels and risk of GSD (*P* < 0.001). Multivariate logistic regression showed that there were weak positive correlations between NAFLD and risk of GSD (*P* = 0.054), and significantly negative correlations between serum HDL-c levels, weight, and hazard of GSD (*P* < 0.01, both), with a weak negative correlation between serum LDL-c and hazard of GSD (*P* = 0.071).

**Table 5:** Univariate and multivariate analysis of gallbladder stone and BMI, WHtR, blood lipid, NAFLD stratified by HC

Factors		Univariate				Multivariate		
	β	OR	95% CI	*P*	β	OR	95% CI	*P*
Gender (M)	−1.411	0.244	0.097–0.612	**0.003**	−0.887	0.412	0.112–1.518	0.183
Age	0.000	1.000	0.968–1.033	0.999		–	–	–
Weight	−0.04	0.961	0.929–0.994	**0.022**	−0.073	0.930	0.875–0.988	0.018
BMI	−0.087	0.917	0.811 ~ 1.036	0.164		–	–	–
WC	0.01	1.010	0.961 ~ 1.062	0.686		–	–	–
WHtR	0.065	1.067	0.987 ~ 1.154	0.105		–	–	–
SBP	−0.007	0.993	0.972–1.015	0.547		–	–	–
DBP	−0.044	0.957	0.919–0.995	**0.029**	−0.044	0.957	0.905–1.011	0.119
FBG	−0.376	0.687	0.485–0.973	**0.034**	−0.206	0.814	0.519–1.277	0.370
TC	0.423	1.526	0.948 ~ 2.457	**0.082**	0.544	1.722	0.818 ~ 3.627	0.153
TG	0.206	1.229	0.916 ~ 1.648	0.169		–	–	–
HDL-c	−1.188	0.305	0.169 ~ 0.548	**< 0.001**	−1.939	0.144	0.055 ~ 0.376	< 0.001
LDL-c	0.401	1.493	1.043 ~ 2.139	**0.029**	−0.641	0.527	0.263 ~ 1.056	0.071
NAFLD	1.002	2.723	1.155 ~ 6.420	**0.022**	1.189	3.284	0.979 ~ 11.013	0.054
Drink	0.189	1.208	0.544 ~ 2.680	0.643		–	–	–

*BMI* body mass index; *WHtR* Waist height ratio; *TG* triglyceride; *HDL-c* high density lipoprotein cholesterol; *LDL-c* low density lipoprotein cholesterol; *NAFLD* nonalcoholic fatty liver disease; *SBP* systolic blood pressure; *DBP* diastolic blood pressure; *FBG* fasting blood glucose; *HC* hypercholesterolemia

- eliminated for no statistical significance in univariate regression

## Discussion

GSD is a common disease that can progress to severe cholecystitis; it is a strong risk factor for gallbladder cancer (GBC). Recent European studies have shown that hypertriglyceridemia, hypercholesterolemia and low levels of high-density lipoprotein cholesterol (HDL) are common in patients with cholelithiasis [23, 24]. To verify the initial hypothesis, the present study performed this clinically-based, age-, sex-, diet-, nation-, blood pressure-, and FBG-matched case-controlled study of the risk factors for asymptomatic GSD, of the epidemiologic factors that are known risk factors for GSD. It was found that elevated BMI, enlarged WC and WHtR, NAFLD, and HC were very likely important risk factors for hazard of new-onset asymptomatic GSD [23–26]. Multivariate logistic regression further strengthened the notion that hypercholesterolemia may be an independent risk of new-onset asymptomatic gallstone diseases, and abdominal obesity and NAFLD might promote the occurrence of asymptomatic gallbladder stone disease.

First, the study confirmed that there were no significant positive correlations between GSD and WC, WHtR after further adjusted by hypercholesterolemia, though obesity has been recognized for its strong association with gallstone diseases. The study showed that obesity per se was not linked directly to cholelithiasis risk, and it was highly probable that both share several pathophysiologic and genomic pathways [27]: On the one hand, hormone-sensitive lipase and adipose triglyceride lipase, discovered recently in adipose tissue, mediate the mobilization of stored triacylglycerol [28]. Elevated triglyceride levels might decrease sensitivity to cholecystokinin [29], and might increase both biliary cholesterol saturation and increase bile viscosity by enhancing mucin production [30], thereby adding to the enhanced risk of gall stone disease. On the other hand, there is a vicious circle: Obesity might result in increasing levels of cholesterol secretion from liver, and bile supersaturation in biliary secretion of cholesterol. Meanwhile, there is a linear relationship between cholesterol production and body fat [31], and biliary cholesterol saturation, bile acid synthesis, turnover rates and bile acid pool sizes are increased in obesity [29, 32]. In addition, it is clear that abdominal adiposity is associated with insulin resistance. Hepatic insulin resistance directly increases cholesterol secretion and reduces bile acid synthesis, leading to bile crystallization and stone formation. Hepatic insulin resistance has been identified as a key determinant of cholesterol gallstones formation by itself [33, 34].

Second, the study showed that the prevalence NAFLD was higher in GSD than in controls, suggesting a positive association between NAFLD and the risk of new-onset asymptomatic gallstone disease. A recent study reported that NAFLD was significantly associated with gallstone formation [35]. It is worth noting that the multivariate regression showed that there was a weak positive correlation between GSD and NAFLD in hypercholesterolemia patients. Based on this observation, it could be speculated that NAFLD, possibly including hypertriglyceridemia, might be a secondary actor in overweight/obesity and hypercholesterolemia patients after eliminating blood pressure and diabetes (blood sugar, insulin resistance). These are the following supporting facts: first, NAFLD is typically characterized by atherogenic dyslipidemia featuring higher triglyceride levels with greater circulating very-low-density lipoprotein levels, lower levels of dense LDL-c and low levels of dysfunctional HDL-c (41). A recent report supported the notion that hypercholesterolemia is a major risk factor for the initiation and development of NAFLD (42), resulting from high-cholesterol diet, lipase deficiencies and liver inflammation (43, 44). Second, NAFLD, which affects approximately 12 to 40% of the general population, up to 95% of obese people, and nearly 70% of the overweight, is especially increased among middle-aged populations with obesity, type 2 diabetes, dyslipidemia, and other metabolic syndromes. Obesity can cause mitochondrial reactive oxygen species (ROS) production and antioxidant depletion in the liver due to higher saturated fatty acid availability and oxidation. Prolonged oxidative stress might favour n-3 long-chain polyunsaturated fatty acid depletion and IR. Liver oxidative stress and insulin resistance (IR) are considered primary abnormalities leading to alterations in hepatic metabolism and the onset of steatosis [36]. NAFLD was observed in 96.5% (*P* < 0.001) of subjects with overweight, hypertriglyceridemia, and hypercholesterolemia [37]. Third, NAFLD is strongly associated with obesity, which might trigger decreased expression of leptin receptor in liver tissue and insulin resistance [38]. The latter two interactions promote the occurrence and development of fatty liver [39]. The prevalence of obesity and NAFLD is undoubtedly closely related to the change of diet structure characterized by an increase in energy intake and the consumption of added sugar and fats. The use of high fructose as sweeteners in beverages has increased significantly. Fructose intake increases the accumulation of fats in the liver and this high fructose intake is considered as one of the components dietary responsible for promoting NAFLD [40]. Fat quantity and quality is in relation to the development of hepatic steatosis. High fat diet and saturated fat may cause hepatic steatosis. Saturated fat changes mitochondrial structure and function and leads to the development and progression of NAFLD [41].

The study showed that hypercholesterolemia may be an independent risk for new-onset asymptomatic gallstone diseases, and recent studies give strong backing to this notion. First, this logistic regression showed that TG and TC were not significantly correlated with GSD in patients with hypercholesterolemia, suggesting that the increased cholesterol and triglycerides play an integrally synergistic role in the pathogenesis of GSD: High levels of secretion of cholesterol in bile and the subsequent supersaturation of bile contribute to the formation of bile mud, increased triglycerides reduce gallbladder movement, reduce the sensitivity to cholecystokinin (CCK), and thereby reduce the gallbladder movement [29, 42]. A recent report strongly supported the notion that sphingolipid ceramide accumulation in hypercholesterolemia causes inflammatory responses of the gallbladder [43]. The experimental observation revealed that significantly increased cholesterol loads lead to imbalances in the state of oxidation and reduction within tissues and ROS accumulation. Excessive ROS can cause lipid peroxidation in cellular membranes, and eventually trigger cell death and tissue damage with the activation of transcription factors such as nuclear factor kappa B (NF-κB) and the generation of oxidized low-density lipoprotein [14, 44]. Second, the study showed that serum LDL-c levels were significant positively associated with the risk of new-onset asymptomatic GSD in participants with hypercholesterolemia. This is in agreement with the findings of Ivanchenkova et al. [45], who found that serum LDL-c levels were increased in gallbladder cholesterosis and were a risk factor for gallbladder cholesterolosis, resulting in the minor particles of LDL more rapidly penetrating into the gallbladder tissue, where the gallbladder wall is intensively involved with macrophages, participating in the formation of foamy cells. Lauridsen et al. [46] found that accelerating elevated LDL-c levels play a synergistic role with the continuous development and formation of gallstones in patients who have undergone cholecystectomy for symptomatic gallstones. Third, a study found that raised HDL-c levels were inversely associated with gallstone prevalence [47], implying that improved HDL-c levels may be one of the mechanisms in the treatment and prevention of cholesterol gallstone disease [48]. Probably, HDL-c, a precursor of bile acids, represents a major source of biliary cholesterol; however, elevated serum HDL increases primary bile acid formation which solubilizes cholesterol and reduces biliary cholesterol saturation [30], both of which are important for reducing the lithogenicity of the bile [47]. HDL might mediate the transport of excess cholesterol from lipid-laden macrophages within the vascular wall back to the liver for excretion into the bile, which represents the major route for irreversible removal of cholesterol from the body [49].

Interestingly, elevated weight was inversely associated with GSD in patients with hypercholesterolemia. The relevant data with the following viewpoints were synthesized: On the one hand, rapid weight loss leads to a change in cholesterol metabolism and consequently increases the concentration of cholesterol in the bile to a level at which not all cholesterol can be dissolved by bile salts. Undissolved cholesterol is prone to crystallize into stones, especially in the presence of calcium and mucin, a glycoprotein that stimulates cholesterol crystal aggregation [50]. On the other hand, weight loss is considered a risk factor for GSD in patients with hypercholesterolemia, resulting from the participants including lean individuals, who may have diet models characterized by irrational vegetarian diets during weight loss. This results in not maintaining reasonably high intake of monounsaturated fats and fiber, olive oil and fish (ω-3 fatty acids), vegetable protein, fruit, coffee, or vitamin C [7, 51]. A recent study showed that a supplementation with 5% chitosan oligosaccharide, a common food additive for weight loss, may cause liver damage via higher hepatic cholesterol accumulation and higher intestinal cholesterol uptake in high fat diet-fed rats [52]. A carbohydrate-restricted, high-fat diet increased LDL-cholesterol concentrations because this effect of weight loss was related to the lack of suppression of both fasting and 24-h free fatty acids [53]. However, weight loss or improper diet may cause imbalance of cholesterol homeostasis, and incomplete and slow gallbladder emptying may lead to cholestasis, increasing the risk of gallstone formation.

### Study strength and limitations

The strength of the study includes a large number of participants, a retrospective design, and complete follow-up for morbidities. The data came from participants who regularly undergo annual physical screening with long-term follow-up. The study reviewed the case histories for more than 5 years, ensuring that the patients had newly presented to the case group. The matched participants had the same occupations and came from similar backgrounds. This healthcare center, the largest in northwest China, is responsible for the health survey of multi-ethnic groups in the adjacent three provinces. The large scale as well as broad coverage of multi-ethnic people made the data very informative and persuasive in this study. Nevertheless, the present study has several limitations. First, this was not a multicenter and cohort study, and the statistics may be biased as a result. Second, the limited ethnic population may affect the generalizability of these findings. In particular, the association between BMI and gallbladder stone may differ among ethnic and regional groups with varying dietary structures and genetic diversity. Third, this study lacked data such as chemical composition analysis, cholesterol gallbladder stone-related risk factors, bile acid levels, and gastrointestinal dysfunction, because imbalance between bile salts and cholesterol in bile fluid turns bile fluid into sludge, crystals and eventually gallstones. Finally, since blood lipids levels varies at different times of the day, one of the limitations of this study is that fasting blood lipid were measured instead of several times during the day.

## Conclusions

Hypercholesterolemia may be an independent risk factor for new-onset asymptomatic gallstone disease. Abdominal obesity and NAFLD might promote the occurrence of asymptomatic gallbladder stone disease. The formation of gallbladder stones is enhanced with constant high concentrations of total cholesterol, which relates to the hypersecretion of cholesterol in the bile and the subsequent supersaturation of bile. This contributes to the formation of biliary sludge. In addition, hypertriglyceridemia consequently reduces gallbladder motility with decreased sensitivity to cholecystokinin. Notably this study first finds that elevated weight was inversely associated with GSD in patients with hypercholesterolemia, suggesting that weight loss or improper diet may cause imbalance of cholesterol homeostasis. Incomplete and slow gallbladder emptying may lead to cholestasis, which increases the risk of gallstone formation. The results of this study suggest that effective control of hyperlipidemia is of greater significance than weight loss, which might make the situation worse, in the prevention of GSD in obese patients with hyperlipidemia.

## Data Availability

The authors declare that the data supporting the findings of this study are available within the article.

## Abbreviations

GSD: Gallbladder stone disease
SBP: Systolic blood pressure
DBP: Diastolic blood pressure
FBG: Fasting blood glucose
BMI: Body mass index
WC: Waist circumference
WHtR: Waist height ratio
TC: Total cholesterol
TG: Triglyceride
HDL-c: High density lipoprotein cholesterol
LDL-c: Low density lipoprotein cholesterol
NAFLD: Nonalcoholic fatty liver disease
HC: Hypercholesterolemia
ROS: Reactive oxygen stress
